# Incidental Parathyroidectomy After Thyroid Surgery: A Single-Center Study

**DOI:** 10.3390/biomedicines12102372

**Published:** 2024-10-17

**Authors:** Roberta Granata, Antonio Zanghì, Marianna Scribano, Giordana Riccioli, Francesca Privitera, Sandro La Vignera, Rosita Angela Condorelli, Francesco Leonforte, Antonio Mistretta, Aldo Eugenio Calogero, Massimiliano Veroux

**Affiliations:** 1 General Surgery Unit, Azienda Policlinico San Marco, University of Catania, 95124 Catania, Italy; robertagranata@live.it (R.G.); amzanghi@unict.it (A.Z.); scribanomarianna@gmail.com (M.S.); giordanariccioli@me.com (G.R.); f.privitera05@gmail.com (F.P.); 2 Department of Clinical and Experimental Medicine, University of Catania, 95124 Catania, Italy; sandrolavignera@unict.it (S.L.V.); rosita.condorelli@unict.it (R.A.C.); acaloger@unict.it (A.E.C.); 3 Hygene Unit, Department of Medical and Surgical Sciences and Advanced Technologies, University of Catania, 95124 Catania, Italy; leonfortefrancesco1@gmail.com (F.L.); anmist@unict.it (A.M.); 4 Vascular Surgery and Organ Transplant Unit, Azienda Policlinico San Marco, University of Catania, 95124 Catania, Italy

**Keywords:** thyroid, multinodular goiter, Hashimoto thyroiditis, neck dissection, hypocalcemia, thyroid cancer, female, thyroid surgery, thyroidectomy, lobectomy

## Abstract

Background: Hypoparathyroidism with hypocalcemia is the most frequent complication after thyroid surgery. Many risk factors have been involved in the development of this complication, with conflicting results. Incidental parathyroidectomy (IP) may be a frequent cause of postoperative hypocalcemia. In this study, we have evaluated the risk factors associated with the IP in a single-center cohort of patients undergoing thyroid surgery. Patients and methods: The incidence and the risk factors for IP were evaluated in 799 patients scheduled for surgical treatment for thyroid disease between January 2018 and December 2023. Parathyroid hormone levels and serum calcium levels, together with the histological specimens, were evaluated in all patients. Results: Post-operative temporary hypocalcemia was present in 239 (29.9%) patients. A total of 144 patients (21.9%) had an incidental parathyroidectomy. Younger patients (<40 years) had the highest risk of having an incidental parathyroidectomy (RR 1.53 (95% CI 1.084–2.161), OR 1.72 (95% CI 1.091–2.710), *p* = 0.014). Moreover, thyroid cancer (RR 1.4 (95 CI 1.114–1.882) OR 1.68 (95% CI 1.145–2.484), *p* < 0.05) and the neck dissection (RR 1.75 (95% CI 1.409–2.198) OR 2.38 (95% CI 1.644–3.460), *p* < 0.001) were strongly associated with the risk of incidental parathyroidectomy. Conclusions: Younger female patients with thyroid cancer and neck dissection were at the highest risk of incidental parathyroidectomy. A meticulous surgical dissection, together with imaging methods for the detection of the parathyroid glands, may reduce the incidence of this complication.

## 1. Introduction

Total thyroidectomy is the most common endocrine surgical procedure performed, and it can lead to serious complications, including vocal cord paralysis or severe bleeding [1]. However, the most common complication following thyroid surgery, causing also a prolonged hospitalization is hypoparathyroidism (hypoPT) [2,3,4]. However, current research findings on the risk factors for post-thyroid surgery hypoPT are not entirely consistent, and the same risk factors may have different impacts on transient and permanent hypoPT [5]. According to a recent meta-analysis, the median incidence of transient and definitive hypoPT ranges from 19 to 38% and 0 to 3%, respectively [6]. The best predictor for identifying hypoparathyroidism is the PTH level monitoring and serum calcium levels [7,8], and the early recognition of hypocalcemia-related symptoms is mandatory for prompt treatment and reduced hospital stay [4,9]. Although there is a lack of agreement about the timing, patient selection, and cut-off points for PTH levels [8,10], most authors demonstrated that PTH levels taken within 1–6 h after skin closure may be predictive of postoperative hypocalcemia and are predictors of early discharge without calcium supplementation [1,6,8,10,11]. Post-operative hypocalcemia can be caused by direct injury, devascularization, the obstruction of venous drainage, or the incidental removal of the parathyroid glands [6,12,13,14]. Indeed, the parathyroid glands are particularly vulnerable to damage during a total thyroidectomy due to their anatomical relationships and shared blood supply [15]. Since the parathyroid glands are tiny and have a similar color to the thyroid, fat, and lymph nodes, it might be challenging to distinguish them from other cervical tissues. Although an accurate surgical technique with a gentle capsular dissection revealing the perithyroidal fatty tissues off the surface of the thyroid may reduce the incidence of incidental parathyroidectomy and parathyroid injury, hypoparathyroidism can be the consequence of a direct injury to parathyroid vascularization [16]. Moreover, many risk factors have been involved in the development of hypoPT, including surgeons’ expertise, the extent of thyroidectomy, hyperthyroidism, malignancy, patient gender, perioperative serum calcium drop, the presence of thyroiditis, and diabetes. Moreover, the number of identified parathyroid glands during surgery can also be considered as risk factors for hypoparathyroidism [1,5,8,11,17,18]. In this study, we have evaluated the risk factors for incidental parathyroidectomy (IP) after thyroid surgery to identify the patient and surgical-related factors that could have a significant correlation with the development of this complication.

## 2. Materials and Methods

This study included all patients who underwent thyroid surgery between January 2018 and December 2023. All patients were scheduled for surgery based either on clinical evaluation or fine needle aspiration. Patients with the affection of calcium homeostasis or patients undergoing completion thyroidectomy were excluded from analysis. Written informed consent, detailing all the surgical procedures and possible complications, was obtained from all patients. Data were retrospectively reviewed and were extracted from a password-protected institutional database, where patients were de-identified and personal information cannot be attributable to the single patient.

Surgical techniques and clinical and follow-up data characteristics have been previously described [11]. Serum calcium, phosphate, and PTH were measured pre-operatively and 6 h after surgery, as previously described [8,11]. Pathology reports were reviewed for the documentation of parathyroids identified in the specimen. Hypoparathyroidism was considered for 6 h PTH levels < 12 pg/mL. Permanent hypoparathyroidism was considered at PTH levels ≤ 12 pg/mL or clinical symptoms of permanent hypocalcemia at 6-month follow-up [8,11]. PTH ratio was calculated by the difference between the preoperative and the postoperative measurements of PTH levels.

Statistical data analysis was performed using SPSS (version 20.0; SPSS Inc. Chicago, IL, USA). Data are expressed as mean ± standard deviation (SD). To compare parametric variables, the Pearson chi-square test or Fisher’s exact test was used. To compare non-parametric variables, Student *t* test or Mann–Whitney U test was used. The difference between the two means was calculated using the Wilson test. Risk ratios (RRs) and odds ratios (ORs) were reported with 95% confidence interval (95% CI) and *p* values. A multivariate analysis, using the risk factors for IP with a *p* value < 0.5 found in univariate analysis, was performed. The level of statistical significance was determined at *p* < 0.05.

## 3. Results

A total of 799 patients underwent thyroid surgery between January 2018 and December 2023. There were 617 women (77.2%) and 182 men (22.8%), with a mean age of 51.4 years (range, 18–75). Patients’ demographics, operative details, histological findings, and postoperative events are reported in Table 1.

Most patients (702, 95.6%) underwent a total thyroidectomy, while 32 patients underwent a lobectomy (4.4%). Multinodular goiter was the most frequent indication for surgery (64.9%), while 178 (24.2%) patients had thyroid cancer.

Post-operative temporary hypocalcemia was present in 239 (29.9%) patients, but most patients presented numbness and tingling in their fingertips, toes, and the perioral region, while no patients presented convulsions. At 6-month follow-up, 18 (2.2%) patients experienced a definitive hypocalcemia.

A total of 144 patients (21.9%) had an incidental parathyroidectomy (Table 2). In 25% of cases, the removed parathyroid gland was intra-thyroidal, suggesting that in these cases, the IP parathyroidectomy seems unavoidable.

Incidental parathyroidectomy was more frequent in female patients (111, 72.1%) undergoing a total thyroidectomy for multinodular goiter (85, 59%) or cancer (50, 34.7%). Younger patients (<40 years) had the highest risk of having an incidental parathyroidectomy (RR 1.53 (95% CI 1.084–2.161), OR 1.72 (95% CI 1.091–2.710), *p* = 0.014) compared to patients > 41 years (OR 0.54 (95% CI 0.402–1.194), *p* = 0.583). Moreover, thyroid cancer (RR 1.4 (95% CI 1.114–1.882) OR 1.68 (95% CI 1.145–2.484), *p* < 0.05), and the neck dissection (RR 1.75 (95% CI 1.409–2.198) OR 2.38 (95% CI 1.644–3.460), *p* < 0.001) were strongly associated with the risk of incidental parathyroidectomy (Table 3). As expected, PTH postoperative levels were significantly lower in patients with unintentional parathyroidectomy (18.3 ± 18.5 vs. 30.3 ± 21, pg/mL, *p* < 0.001), with a significant higher incidence of postoperative biochemical hypocalcemia (44.4% vs. 35.5%, *p* = 0.042), and the PTH ratio was significantly lower in patients with IP, suggesting a greater reduction in PTH levels as a consequence of IP. However, the 35.5% of patients without unintentional parathyroidectomy had a transitory hypocalcemia, suggesting that the direct injury to the parathyroid during the surgical dissection may have a relevant role in the development of hypocalcemia and hypoPT. Moreover, the risk of definitive hypoparathyroidism was higher in patients with unintentional parathyroidectomy (8, 5.6%) compared with those without (11, 1.7%, *p* = 0.032): however, at 6-month follow-up, there was not a significant difference in serum calcium levels between the two groups. Interestingly, the presence of a multinodular goiter, Hashimoto thyroiditis, and a larger thyroid volume did not correlate with an increased risk of incidental parathyroidectomy.

## 4. Discussion

The growing incidence of thyroid disease made hypocalcemia and hypoparathyroidism a frequent complication of thyroidectomy [19,20,21]. The mechanism underlying the development of this condition is not precisely disclosed, although it is accepted to be multifactorial [1,17,18,22]. During the past years, many studies have evaluated the likely risk factors for post-thyroidectomy hypocalcemia, yet establishing these risk factors with certainty continues to be a challenge. In our study, the IP was reported in 144 patients (21.9%). The extent of surgery may in principle increase the risk of IP: Ying-hao Wang et al. [19] found that the near-total thyroidectomy and total thyroidectomy were independent risk factors for postoperative hypoparathyroidism [19]. Moreover, other studies suggested that the total thyroidectomy was associated to up to a 6.5-fold higher prevalence of permanent hypoparathyroidism in patients with benign thyroid diseases [23] when compared to near-total thyroidectomy or lobectomy [23,24]. Interestingly, completion thyroidectomy may be associated with an increased risk of IP compared to primary thyroidectomy [25,26]. However, a recent meta-analysis reported limited evidence that the extent of surgery might influence the risk of transient or permanent hypocalcemia [27]. This observation was confirmed in our study, since the incidence of IP was not statistically different between patients undergoing total thyroidectomy compared with those undergoing lobectomy: this could be related to the low number of lobectomies performed in our center and that most of these were for benign disease. Indeed, thyroid cancer and neck dissection may increase the risk of IP: we have previously reported that patients with thyroid cancer undergoing a neck dissection have an increased risk of incidental parathyroidectomy with the highest risk for >8 lymph nodes retrieved [8,11]. This was confirmed by other authors who found a higher incidence of hypoparathyroidism in patients undergoing neck dissection or lateral lymph node dissection [5,11,28,29]. This increased risk is probably higher because the parathyroid glands are at risk of being removed incidentally during central compartment dissection [30], and, because in patients who underwent extensive resections and had a clinical N1a disease, the inferior glands were more frequently involved in incidental parathyroidectomy after total thyroidectomy [31]. In contrast, Barrios et al. [32] and Lin et al. [33] did not find an association between the central node dissection and the IP [32], although some authors [30,31,34] reported an increased risk of IP in patients with thyroid cancer, probably because of cancer invasion to the parathyroid gland along with adjacent tissue. It should be expected that direct visualization of the parathyroid gland during thyroid surgery may reduce the incidence of IP: however, Riordan et al. [35] found that the number of parathyroid glands identified during thyroid surgery is directly correlated with an increased risk of postoperative hypocalcemia, and this might raise the question as to whether the dissection of parathyroid glands during identification itself is a risk factor for postoperative dysfunction. However, different studies suggest that a more meticulous intraoperative identification of the parathyroid gland might reduce the incidence of incidental parathyroidectomy since 16.7–40.0% of the incidentally excised parathyroid glands were found in an intracapsular location and 15.7–81.1% were found in an extracapsular location [30,33]. On the other hand, the number of identified parathyroid glands during surgery may not influence the occurrence of hypocalcemia [12], and, in cases where the parathyroid glands are in intrathyroidal position, unintentional parathyroidectomy is inevitable, even in the hands of an experienced surgeon [36,37,38,39], as confirmed in our experience where 25% of the excised glands were intra-thyroidal. To increase the detection of parathyroid glands during surgery, several non-invasive techniques have been proposed, including the fluorescence-image-guided thyroid surgery, which could significantly increase the rate of the detection of parathyroids, differentiating them from lymph nodes, thereby reducing the risk of IP [40], and could be particularly beneficial for surgeons with less experience in the field. Near-infrared autofluorescence may have a potential in decreasing the risk of hypoPT, although its sensitivity and sensibility in detecting the parathyroid glands is increased only when used in combination with the indocyanine green fluorescence [41]. Female gender and younger age were reported to increase the rate of IP with conflicting results, and our study showed that female patients had an increased risk of IP and hypoparathyroidism. Our findings show that 25–40% of female patients may develop temporary postoperative hypocalcemia compared to 10% of male patients [9,12,42]. There are many suggested explanations for this increased risk. Firstly, female patients often present with more severe thyroid conditions preoperatively, such as hyperthyroidism or cancer [33,34,41,43], and this could increase the thyroid surgery complexity and the likelihood of parathyroid injury compared to males. Secondly, the gender different levels in PTH and vitamin D can affect postoperative parathyroid function recovery [6,41]: moreover, thyroid surgery may disrupt hormone balance, particularly in menopause women, thereby increasing the risk of postoperative hypoparathyroidism. Indeed, estrogen may influence parathyroid function and promote calcium absorption in the intestines, thereby contributing to the maintenance of stable serum calcium levels [5]. Lastly, the stronger immune responses observed in females may affect postoperative inflammation and healing processes, finally increasing the risk of IP [33,34,41,43]. This was confirmed by a recent meta-analysis [27], which found that female patients with thyroid cancer undergoing a neck dissection have the highest risk for the development of hypoPT. Yann-Sheng Lin et al. [30] confirmed that sex and gender are not independent factors for IP, even though the IP group’s mean patient age was higher than the age of the non-IP group.

On the other hand, McMurran et al. [27] have demonstrated that post-thyroidectomy hypocalcemia is not consistently correlated with the age or sex of the patients [27]. This is supported also by other authors who, even though registered a higher number of female patients developing protracted hypoparathyroidism, the difference was not statistically significant [44]. The influence of age in the development of post-thyroidectomy hypoparathyroidism is still a conflicting topic. In our study, we observed a positive correlation between younger (<40 years old) female patients and the development of hypoparathyroidism. While some studies support our findings [11,37,45,46,47], probably related to a more aggressive surgical approach in younger patients for malignancies [46], on the other hand, Sousa et al. [48] reported that patients older than 50 years were 1.9 times more likely to present with hypocalcemia than younger patients: moreover, other data suggest that the incidence of IP increases in patients older than 60 years [49], while Del Rio et al. [1] divided the patients into four age groups and found no significant intergroup difference, which is in agreement with other observations made by Edafe et al. [6].

In this study, the large thyroid volume and thyroiditis did not correlate with a higher risk of IP, as reported in many studies that did not find a direct relationship between the presence of a large multinodular goiter and the risk of IP [5,27,33,50].

The association between IP and postoperative hypocalcemia is still debated, and it is variable according to the different definitions of transient/ definitive hypocalcemia. In this series, the IP was associated with an increased risk of both temporary and permanent hypocalcemia. These findings were confirmed by many authors [5,11,27,30,33] who found a significant association between IP and postoperative hypocalcemia. To give a more precise definition of the risk of hypocalcemia in patients with IP, Bai et al. [33], in their interesting review, evaluated the incidence of temporary clinical and biochemical hypocalcemia in patients with IP: their analysis confirmed that the incidence of clinical hypocalcemia (4–39%) was lower than that of biochemical hypocalcemia (3–56%), but, interestingly, IP was found to be associated with an increased incidence of temporary/permanent hypocalcemia, regardless of whether the biochemical or clinical definition was used [33]. Interestingly, in studies with low IP incidence, IP increased the risk of temporary hypocalcemia but not of definitive hypocalcemia [33], suggesting that the surgeon experience may play a fundamental role in the incidence of IP during thyroid surgery. This study investigated also the PTH ratio and found a significantly lower ratio in patients with IP, suggesting that a greater reduction of PTH levels might be expected in this group of patients. Some studies provided evidence that a reduction of PTH levels greater than 62% is associated with a sensitivity and specificity of 100% to develop a transient hypocalcemia within postoperative day 2 [21].

This study has some limitations: the study sample is relatively small, although it includes a single-center experience, thus reducing the biases related to multicentric studies. In this study, no method of the intraoperative detection of the parathyroid glands has been used, and this may have potentially increased the rate of postoperative hypoPT. Finally, the influence of surgeon volume on postoperative hypoparathyroidism has been extensively explored in the literature, showing that a surgeon annual operative value > 50 thyroid operations is associated with the lowest risk of hypoparathyroidism [51]: however, we did not evaluate the influence of the surgeon’s experience on the rate of incidental parathyroidectomy since all thyroid surgeries were performed by the same surgical team.

In conclusion, thyroid surgery might be associated with a potential high rate of incidental parathyroidectomy. The risk factors involved in the development of this condition are female sex, younger age, thyroid cancer, and neck dissection, while we found no correlation with the extent of the thyroid surgery. As hypoparathyroidism is in most cases related to surgical procedure, it is advisable to perform a scrupulous dissection of the thyroid gland to minimize the risk of this complication.

## Figures and Tables

**Table 1:** Patients’ characteristics of the entire cohort (n = 799).

Characteristic	*N* (%)
**Gender**	
Female	617 (77.2)
Male	182 (22.8)
**Mean age (years)**	51.4 ± 17.8
**Surgical Procedure**	
Total thyroidectomy	761 (95.2)
Lobectomy	38 (4.8)
**Histological Diagnosis**	
Multinodular goiter	535 (76.5)
Hashimoto thyroiditis	126 (15.7)
Thyroid cancer	178 (24.2)
**Unintentional parathyroidectomy**	144 (20.6)
Portion of parathyroid	18 (22.5)
One parathyroid	126 (87.5)
Intrathyroidal parathyroid	36 (25)
Perithyroidal parathyroid	108 (75)
**Parathyroid glands identified during surgery**	
0	20 (2.5)
1	49 (6.1)
2	211 (26.4)
3	326 (40.8)
4	193 (24.1)
**Auto-transplanted parathyroids**	32 (4.0)
**Post-operative hypocalcemia**	239 (29.9)
**Definitive hypocalcemia**	18 (2.2)

**Table 2:** Risk factors for unintentional parathyroidectomy.

	Unintentional Parathyroidectomy	No Parathyroidectomy	
Characteristics	*N* (%)	*N* (%)	*p* Value
**Patients**	144 (21.9)	655 (77.9)	
**Sex**			
Male	33 (22.9)	149 (29.4)	0.532
Female	111 (72.1)	506 (70.6)	0.543
**Age (years, %)**	50.7 ± 14.5	53.6 ± 11.9	<0.05
Age Groups			
<40	36 (25)	112 (17.1)	0.010
40–55	47 (32.6)	215 (32.8)	0.428
>55	61 (42.4)	328 (50.1)	0.645
**Total thyroidectomy/lobectomy**	139 (96.5)	622 (95)	0.824
**Lobectomy**	5 (4.5)	33 (5)	0.885
**Neck dissection**	65 (45.1)	168 (25.7)	<0.001
**Preoperative PTH (mean, pg/mL)**	54 ± 23.6	54.8 ± 20	0.224
**Postoperative PTH (mean, pg/mL)**	18.4 ± 21.0	30.3 ± 21	<0.001
**PTH ratio**	65.7%	44.5%	<0.05
**Temporary hypocalcemia < 8 mg/dL**	64 (44.4)	233 (35.5)	0.042
**Definitive hypocalcemia**	8 (5.6)	11 (1.7)	0.032
**Underlying disease**			
Cancer	50 (34.7)	157 (24)	0.018
Multinodular goiter	85 (59)	450 (68.7)	0.045
Hashimoto thyroiditis	23 (15.9)	103 (15.7)	0.632
Others	2 (1.4)	3 (1.4)	0.632
**Thyroid volume (mean, cm^2^)**	26.9 ± 22	34.3 ± 29.2	0.01
**Postoperative serum calcium (mean, mg/dL)**	8.2 ± 0.45	8.3 ± 0.7	0.122
**6-month postoperative serum calcium (mean, g/dL)**	9.2 ± 0.45	9.6 ± 0.35	0.233

**Table 3:** Multivariate analysis of risk factors for incidental parathyroidectomy.

Characteristics	OR	95% CI	*p* Value
**Age**			
>40 y	1		
<40 y	1.53	1.084–2.161	<0.001
**Multinodular goiter**	0.81	0.693–0.952	0.322
**Neck dissection**	1.75	1.409–2.198	<0.001
**Thyroid cancer**	1.4	1.114–1.882	0.022

## Data Availability

De-identified data can be made available upon reasonable request.
